# Use of ATP-Binding Cassette Subfamily A Member 13 (ABCA13) for Sensitive Detection of Focal Pathological Forms of Subclinical Bovine Paratuberculosis

**DOI:** 10.3389/fvets.2022.816135

**Published:** 2022-03-10

**Authors:** Cristina Blanco-Vázquez, Marta Alonso-Hearn, Natalia Iglesias, Patricia Vázquez, Ramón A. Juste, Joseba M. Garrido, Ana Balseiro, María Canive, Javier Amado, Manuel A. Queipo, Tania Iglesias, Rosa Casais

**Affiliations:** ^1^Centro de Biotecnología Animal, Servicio Regional de Investigación y Desarrollo Agroalimentario (SERIDA), Deva, Spain; ^2^Department of Animal Health, NEIKER-Basque Institute for Agricultural Research and Development, Basque Research and Technology Alliance (BRTA), Derio, Spain; ^3^Departamento de Sanidad Animal, Facultad de Veterinaria, Universidad de León, León, Spain; ^4^Instituto de Ganadería de Montaña, Centro Superior de Investigaciones Científicas (CSIC-Universidad de León), León, Spain; ^5^Laboratorio Regional de Sanidad Animal del Principado de Asturias, Gijón, Spain; ^6^Servicio de Sanidad y Producción Animal del Principado de Asturias, Oviedo, Spain; ^7^Unidad de Consultoría Estadística, Servicios científico-técnicos, Universidad de Oviedo, Gijón, Spain

**Keywords:** paratuberculosis, subclinical infection, focal lesions, diagnosis, biomarkers, ATP binding cassette subfamily A member 13

## Abstract

Bovine paratuberculosis (PTB) is a chronic enteritis caused by *Mycobacterium avium* subspecies *paratuberculosis* (Map) that causes a heavy economic impact worldwide. Map infected animals can remain asymptomatic for years while transmitting the mycobacteria to other members of the herd. Therefore, accurate detection of subclinically infected animals is crucial for disease control. In a previous RNA-Seq study, we identified several mRNAs that were overexpressed in whole blood of cows with different PTB-associated histological lesions compared with control animals without detected lesions. The proteins encoded by two of these mRNAs, ATP binding cassette subfamily A member 13 (ABCA13) and Matrix Metallopeptidase 8 (MMP8) were significantly overexpressed in whole blood of animals with focal histological lesions, the most frequent pathological form in the subclinical stages of the disease. In the current study, the potential of sensitive early diagnostic tools of commercial ELISAs, based on the detection of these two biomarkers, was evaluated in serum samples of 704 Holstein Friesian cows (566 infected animals and 138 control animals from PTB-free farms). For this evaluation, infected animals were classified into three groups, according to the type of histological lesions present in their gut tissues: focal (*n* = 447), multifocal (*n* = 59), and diffuse (*n* = 60). The ELISA based on the detection of ABCA13 was successfully validated showing good discriminatory power between animals with focal lesions and control animals (sensitivity 82.99% and specificity 80.43%). Conversely, the MMP8-based ELISA showed a poor discriminatory power between the different histological groups and non-infected controls. The ABCA13-based ELISA showed a higher diagnostic value (0.822) than the IDEXX ELISA (0.517), the fecal bacterial isolation (0.523) and the real-time PCR (0.531) for the detection of animals with focal lesions. Overall, our results indicate that this ABCA13 ELISA greatly improves the identification of subclinically infected animals with focal lesions that are undetectable using current diagnostic methods.

## Introduction

Bovine paratuberculosis (PTB) or Johne's disease (JD) is a chronic contagious and debilitating enteritis of domestic and wild ruminants caused by *Mycobacterium avium* subspecies *paratuberculosis* (Map). PTB produces important economic losses in dairy herds worldwide due to increase in mortality, decrease in milk production, weight loss, premature culling and reduced slaughter value (1–4). PTB has also been related to reduced fertility rates (5, 6) and increased susceptibility to other diseases, particularly mammary infections (7). The importance of this disease would be even greater when considering its zoonotic potential and the risk of transmission of viable Map through pasteurized milk and milk products (8–10). The association of Map with human diseases like Crohn's disease (CD), type I diabetes (T1D), multiple sclerosis (MS) or rheumatoid arthritis (RA) has been documented (11–16).

The main clinical signs of the disease are progressive weight loss, diarrhea, and decreased milk yield (17). Map typically enters a herd through the purchase of subclinically infected cattle shedding the bacteria with feces that contaminate the environment, although it can also enter through contaminated feces adhering to vehicles, equipment, and visitors. Access to contaminated pasture or water sources and contact with other ruminants may also be involved in the spread of the disease (18–20).

Different pathological forms associated with Map infection can be established: focal, multifocal and diffuse (21). Briefly, focal lesions consist of small scattered and well-demarcated granulomas composed by macrophages and few Langhans giant cells, mainly located in the jejunal and ileal lymph nodes and not affecting the intestinal lamina propria. Multifocal lesions consist of numerous well-demarcated granulomas in the intestinal lymphoid tissues and in the intestinal lamina propria. Diffuse lesions are characterized by extensive severe and diffuse granulomatous enteritis and lymphadenitis, that markedly alter the normal histological structure. According to the inflammatory cell type present in the infiltrate and the number of acid-fast bacilli (AFB), diffuse lesions were further subdivided into diffuse lymphoplasmocytic paucibacillary, diffuse intermediate and diffuse histocytic multibacillary lesions (22). Three epidemiopathogenic forms of the infection (apparently free, latent and patent) have been described according to the immunopathological classification, epidemiological aspects and diagnostic results (23). The latent forms show no clinical signs and are characterized by a low bacterial load, a predominant cell-mediated immune response and the presence of mild histological lesions in the intestine and associated lymph nodes. The patent forms are characterized by a variable degree of signs associated with advanced multifocal and diffuse lesions. Apparently free PTB animals are those negative by all diagnostic tests not showing any visible microscopic lesions.

Although vaccination has been shown to be an efficient tool (24, 25) and is widely practiced in some settings (26), most PTB control programs in cattle are based on testing and culling test-positive cows combined with good management practices (27). However, the efficiency of the control programs based on the “test and cull” policy is strongly conditioned by the diagnostic methods used to detect Map infection, the performance of which varies depending on the stage of the Map infection. Current diagnostic tests have low sensitivities to detect subclinical infections. Consequently, novel diagnostic tools with high sensitivity and specificity would improve the diagnostic efficacy necessary to control the disease. The potential of emerging -omic approaches to complement and enhance the diagnosis of Map infection in cattle has been previously reviewed (28) and point out the potential of host biomarkers as tools to develop novel diagnostic methods for PTB (29–34). In a previous study, whole RNA-sequencing (RNA-Seq) identified host genes differentially expressed in peripheral blood samples collected from animals with focal or diffuse lesions in gut tissues *versus* (*vs*.) control animals without any detected lesion (35). Genes encoding for bovine proteins MMP8 (Matrix metallopeptidase 8) and ABCA13 (ATP binding cassette subfamily A member 13) showed significantly higher expression in animals with focal lesions (log2 fold change 1.89 and 3.74, respectively), while genes encoding for FAM84A (family with sequence similarity 84 member A), SPARC (secreted protein acidic and cysteine rich) and desmin (DES) were up-regulated in animals showing diffuse lesions when compared to control animals (log2 fold change 2.15, 2.21 and 3.75, respectively). Subsequently, the diagnostic performance of commercial ELISAs based on the detection of those five blood candidate biomarkers to detect animals with focal, multifocal and diffuse lesions was studied (*n* = 155) and compared to that of conventional PTB diagnostic methods (IDEXX ELISA and feces and tissues real-time polymerase chain reaction (PCR) and bacteriological culture) (36). The results indicated that the biomarker-based ELISAs consistently showed higher sensitivity values than the conventional immunologic and microbiologic diagnostic methods. The ABCA13-based ELISA had the best diagnostic performance for global detection of animals with any type of lesions (69.41% sensitivity *vs*. 28.41%, 11.36%, and 25% of the IDEXX ELISA, fecal culture and fecal PCR, respectively) improving overall detection of animals with PTB-specific histological lesions. Most importantly, the ABCA13-based ELISA showed the most accurate diagnostic performance for detection of animals with focal lesions, the most frequent pathological form in the subclinical stages of the disease, with a 79.25% sensitivity *vs*. the 14.55%, 5.45%, and 9.09% sensitivities of the IDEXX ELISA, fecal culture and fecal PCR, respectively. The MMP8-based ELISA showed the highest diagnostic accuracy for global detection of animals with multifocal and diffuse lesions with a sensitivity of 96.97% *vs*. the 54.44%, 51.51%, and 21.21% of the IDEXX ELISA, fecal culture and fecal PCR, respectively.

ATP-binding cassette proteins are a superfamily of transporter proteins that play important physiological roles in living organisms and are vital for normal brain function (37–39). Through coupling the energy of ATP hydrolysis, they translocate solutes such as lipids, ions, peptides and xenobiotics through biological membranes (38, 40), but they also intervene in a wide variety of other processes such us signal transduction, protein secretion, drug and antibiotic resistance, antigen presentation and bacterial pathogenesis and sporulation (41). Within this superfamily of proteins, there is the subfamiliy ABCA, functionally characterized by shuttling lipid molecules across cell membranes (42). Disruption to ABC transporter activity results in lipid accumulation and an elevated level of inflammatory cytokines in lung tissue (43). One of the most polymorphic genes of this subfamily is ABCA13, with 165 alterations described (44). ABCA13 is expressed in several human tissues and is involved in several pathologic and physiologic processes in the human body (45). Thus, it has been associated with neurological disorders such as major depressive disorder (46), Lewy body dementia (47), schizophrenia and bipolar disorder (48), and autism spectrum disorder (49, 50). Conversely, ABCA13 has also been linked to cancer, sometimes associated with a poor prognosis (51–55) and on other occasions with a longer patient survival (56, 57). ABCA13 is also considered a useful marker for predicting lymph node metastasis in resected gastric cancer patients in the early stage (54). Among its related pathways are CDK-mediated phosphorylation and removal of Cdc6 and Innate Immune System. Gene Ontology (GO) annotations related to this gene include ATPase activity and cholesterol transporter activity. In cattle, the ABCA13 gene has been related to osteogenesis imperfecta, the origin of this disease being attributed to the additive effects of this gene with QRFPR and IFIM5 (58).

Matrix metalloproteinases (MMPs) are metal-dependent enzymes that degrade components of the extracellular matrix and other components such as receptors, growth factors, cytokines and chemokines (59–61) and that also play an important role as effective regulators of cell proliferation and differentiation, tissue homeostasis and immune response (62) via signal transduction in immune cell signaling (63). Some MMPs are related to cancer progression, metastasis and invasion (64, 65) through the induction of changes during the epithelial-mesenchymal transition in the tumor microenvironment (66). The MMPs family includes collagenases, gelatinases, stromelysins, membrane type MMPs (MT-MMPs), matrilysins and various other MMPs (67), whose activity is tightly regulated by a family of endogenous inhibitors termed as tissue inhibitors of metalloproteinases (TIMPs) (68). Twenty-eight forms of MMPs have been identified in vertebrates and 24 in humans (69). Among them, metalloproteinase 8 (MMP8) or neutrophil metalloproteinase, an enzyme released by neutrophils and by other cells of the non-polymorphonuclear lineage such as gingival fibroblasts, bone and plasma cells (70) and that breaks down type I, II, III, V, VII, VIII and X collagen, gelatin, aggrecan, elastin, fibronectin, laminin and nidogen (71) which plays an important role in cell proliferation, migration, and angiogenesis through the development of capillary-like network structures (72). MMP8 is involved in tumor survival and morbidity since its expression has been associated with pro- and anti-inflammatory effects in different types of tumor microenvironments (73). Thus, sometimes it is related to a worse outcome of certain cancers (72, 74–76) although some authors defend its protective role (77). There is evidence suggesting that Map can induce the expression of MMPs, which are the main proteases in the pathogenesis of inflammatory bowel disease (78). TIMSPs have been suggested as potential biomarkers for tuberculosis (TB). TIMPs-1,−2 and−3 facilitate remodeling and repair of tissue following destruction by MMPs. For instance, the concentration of MMP8 has been demonstrated to decrease rapidly during TB treatment (79). MMP9 and TIMP1 are known to be up-regulated in TB infection and have been proposed as biomarkers for diagnosis of TB (80).

Two candidate biomarkers, ABCA13 and MMP8, were selected for further validation, based on the results obtained in the RNA-SEQ analysis (35) and in the pre-validation study (36). The aim of the present study was to carry out a robust validation of the diagnostic potential of ELISAs based on detection of ABCA13 and MMP8, using reference serum samples from well characterized animals, classified according to the different pathological forms associated with Map infection [focal (*n* = 447), multifocal (*n* = 59), and diffuse (*n* = 60)], and control animals from PTB-free farms (*n* = 138).

## Materials and Methods

### Animals and Samples

A total of 704 Holstein-Friesian cows, with an average age of 5.2 years ranging from 0.5 to 13.4, were included in this study. The positive reference group consisted of 566 cows from 8 different regions of Spain (Basque Country, Catalonia, Navarra, Aragón, Cantabria, Castilla y León, La Rioja and Asturias) commercially slaughtered in five abattoirs located in the Basque Country and Asturias (23, 36, 81). The negative reference group consisted of 138 live cows from 4 PTB-free farms of Asturias.

Samples of blood and feces were collected from all the animals while tissue samples (ileocecal valve, caudal ileum, and caudal jejunal and ileal lymph nodes) were only taken from the slaughtered animals, *in situ* after evisceration either in the Asturian abattoirs or in the necropsy room at NEIKER. Tissue samples were split for molecular, microbiological, and histopathological analysis.

Additionally, in order to assess the analytical specificity of the ABCA13-based ELISA in relationship with other mycobacterial infections, two sets of samples from animals infected with *Mycobacterium bovis*, the causative etiological agent of TB, were used. One bovine set consisted of 30 samples from experimentally infected cows, 10 sera collected at 8- and 12-weeks post-infection and 20 plasma samples taken at 16 weeks post-infection. The other set consisted of 27 sera from cows naturally infected both with a positive result in the comparative intradermal tuberculin test and a negative result in the IDEXX ELISA for the detection of Map-specific antibodies.

### Assessment of Map Infection Status of Positive and Negative Reference Animals

Tissue samples from positive reference animals were processed as described in (36) and classified according to the type and extension of the histological lesions present in their intestinal tissues and associated lymph nodes into three groups according to González et al. (21) (see Supplementary Figure 1): focal (*n* = 447, 78.98%), multifocal (*n* = 59, 10.42%), and diffuse (*n* = 60, 10.60%). The Map infection status of these 566 positive reference animals was further characterized (Table 1) by serology (IDEXX Map Ab test –IDEXX, Montpellier, France), bacteriological isolation and real-time PCR of tissues and feces (in some cases), following procedures previously described (23, 36). The percentage of positive animals by one or more diagnostic techniques increased in parallel with the severity of the lesions. Thus, the percentage of positive animals for at least one of the techniques in the focal, multifocal and diffuse groups was 31.99%, 88.14%, and 100%, respectively. More specifically, in the group of animals with focal lesions, 3.36 % were positive by serum IDEXX ELISA and 15.51, and 16.85% were positive by fecal culture and real-time PCR, respectively. In the group of animals with multifocal lesions, 30.51 % were positive by serum IDEXX ELISA and 50.85% and 59.32% were positive by fecal culture and real-time PCR, respectively. In the diffuse group, 86.67% of animals were positive by serum ELISA and 85 and 90% were positive by fecal culture and real-time PCR, respectively. In the same way, the percentage of animals positive by all diagnostic techniques increased as we moved from the focal to the diffuse group, being 0.89%, 8.0%, and 13.64% for the focal, multifocal and diffuse group, respectively. The PTB-free status of the negative reference animals from farms with no prior history of clinical disease was verified yearly by repeatedly negative IDEXX serum ELISA results during a period of 3 to 5 years depending on the specific farm (2016–2021). In one of the farms, the PTB-free status (*n* = 61) was also confirmed by fecal real-time PCR and bacterial isolation.

**Table 1:** *Mycobacterium avium* subspecies *paratuberculosis* (Map)-infectious status of the 566 infected animals according to the reference histopathological classification (21).

**Diagnostic technique**	**Histopathological type**		
	**Focal (*n* = 447)**	**Multifocal (*n* = 59)**	**Diffuse (*n* = 60)**
Bacteriological culture (feces)	3/65 (4.62%)	3 (12%)	3 (13.64%)
Bacteriological culture (tissues)	69/445 (15.51%)	30 (50.85%)	51 (85%)
Bacteriological culture (both)	1/65 (1.54%)	2 (8%)	3 (13.64%)
Real-time PCR (feces)	4/65 (6.15%)	10 (40%)	17 (77.27%)
Real-time PCR (tissues)	75/445 (16.85%)	35 (59.32%)	54 (90%)
Real-time PCR (both)	4/65 (6.15%)	8 (32%)	16 (72.77%)
IDEXX serum ELISA	15/447 (3.36%)	18 (30.51%)	52 (86.67%)
Ziehl Neelsen stain	76/445 (17.08%)	45 (75.59%)	60 (100%)
Positive to at least one diagnostic test	143 (31.99%)	52 (88.14%)	60 (100%)
Positive to all diagnostic tests	4 (0.89%)	2 (8%)	3 (13.64%)

*Real-time PCR, real-time polymerase chain reaction. Percentages of positivity obtained for each technique are shown in parentheses. Since not all the diagnostic techniques were performed in all the animals, the percentages of positivity were calculated as the number of positive animals divided by the total number of animals analyzed by that specific technique. The Ziehl Neelsen stain is considered a confirmatory technique of fast acid bacteria, not a specific diagnostic method of PTB*.

### Enzyme-Linked Immunosorbent Assay (ELISA) for Biomarkers Detection

The ABCA13 and MMP8 serum concentration was measured using commercially available Enzyme-Linked Immunosorbent Assays (ELISAs) according to the manufacturer's instructions (MyBioSource, San Diego, CA. USA). Competitive Bovine ATP-binding cassette subfamily A member 13 ELISA kit (detection range 1–5,000 pg/mL) and Bovine Matrix Metalloproteinase 8 (detection range 3.12–100 ng/mL) were used for specific detection of ABCA13 and MMP8 bovine biomarkers, respectively. According to the manufacturer, a standard curve was used to determine the concentration of these biomarkers in the serum samples. A 4-parameter MMF equation provided the best fitting between the average Define optical density (OD) of each standard dilution and its concentration. Standards and blanks were tested in duplicate but a single well was used with test samples. The mean value of the blank control was subtracted from the raw OD values before result interpretation. The concentration of ABCA13 and MMP8 in each sample was interpolated from the standard curve.

### Statistical Analysis

Data obtained from ELISA quantification was analyzed using the pROC and Optimal Cutpoints packages of R Program Statistical environment version 3.6.0 (http://www.Rproject.org/), with confidence intervals (CI) stated at 95% for the final results. The area under the curve (AUC) and optimal cut-off value for each group was determined individually by Receiver operator characteristic (ROC) curve analysis. The optimal cut-off values for sensitivity and specificity were based on maximum Youden Index (J = Se + Sp-1). The discriminatory power of this ELISA-based ABCA13 to discern between the different histopathological groups and the control group was considered as follows: AUC values ≥0.9 were considered to have excellent discriminatory power; 0.8 ≤ AUC <0.9 good discriminatory power; 0.7 ≤ AUC <0.8 fair discriminatory power; and AUC <0.7 poor discriminatory (78, 82).

The model developed by Juste and Casal (83) was fed with the sensitivity and specificity of the different testing methods used in this study in order to compare their performance for PTB control in defined epidemiological circumstances.

## Results

### Diagnostic Performance of the ABCA13 and MMP8 Biomarker-Based ELISAs

**Figure 1:**
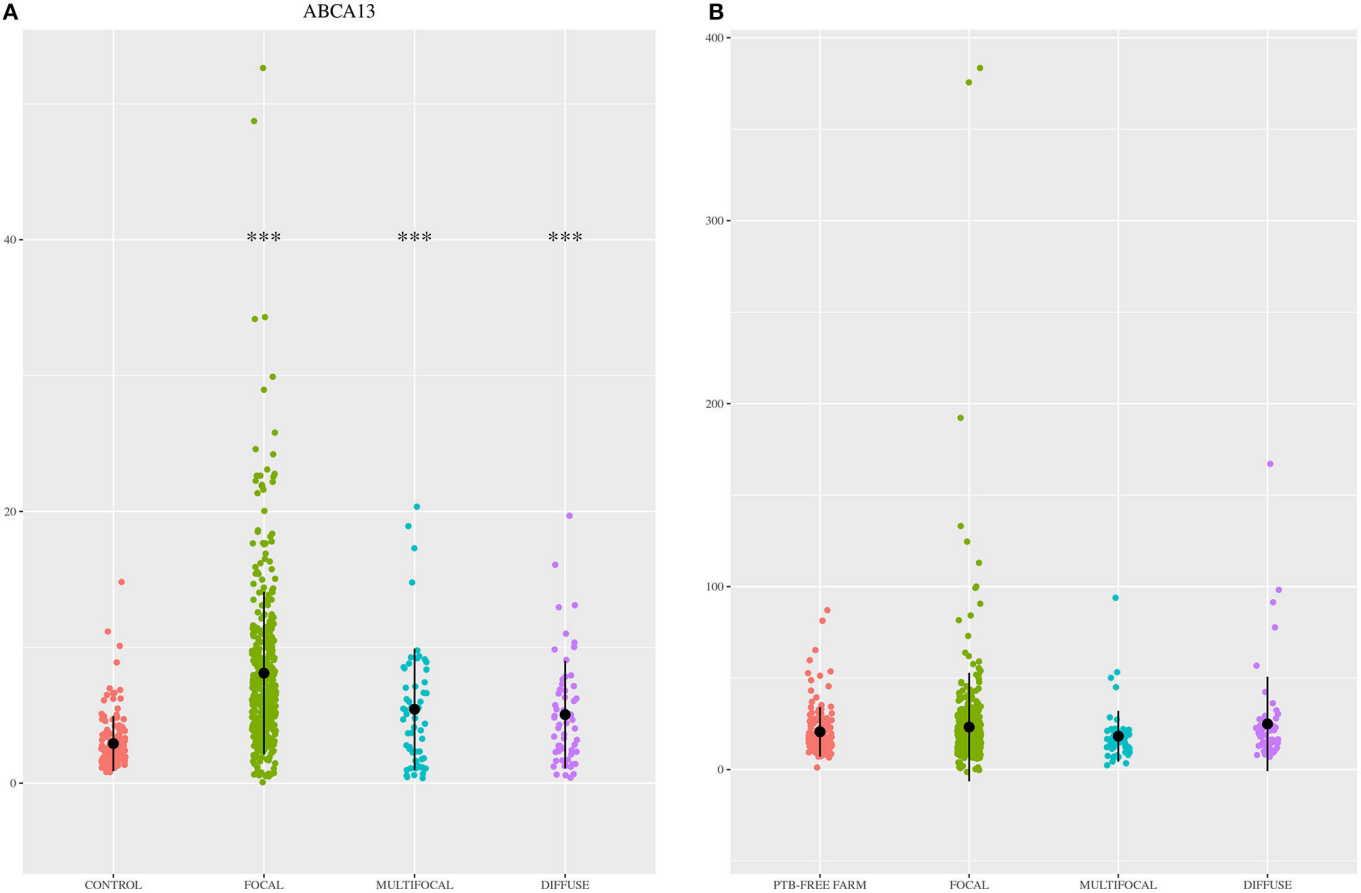
ABCA13 and MMP8 expression levels in the 704 animals included in the study. **(A)** ABCA13 expression levels in sera of Holstein Friesian cattle showing different types of histological lesions consistent with paratuberculosis (PTB) in their intestinal tissues [focal (*n* = 447), multifocal (*n* = 59), and diffuse (*n* = 60)] and control animals from PTB-free farms (*n* = 138). **(B)** MMP8 expression levels in serum of Holstein Friesian cattle showing different types of histological lesions consistent with PTB in their intestinal tissues [focal (*n* = 442), multifocal (*n* = 58), and diffuse (*n* = 60)] and control animals from PTB-free farms (*n* = 138). Biomarkers were quantified by specific ELISAs supplied by MyBioSource, San Diego, CA, USA; ABCA13, bovine ATP binding cassette subfamily A member 13; MMP8, bovine matrix metallopeptidase 8. The data are represented as scatter plots with each dot representing a single animal. The mean of each histopathological group is represented by a gross black point and the standard deviation by a vertical line. The asterisks indicate whether differences between each histopathological group and the control are or not significant (***p < 0.001).

**Figure 2:**
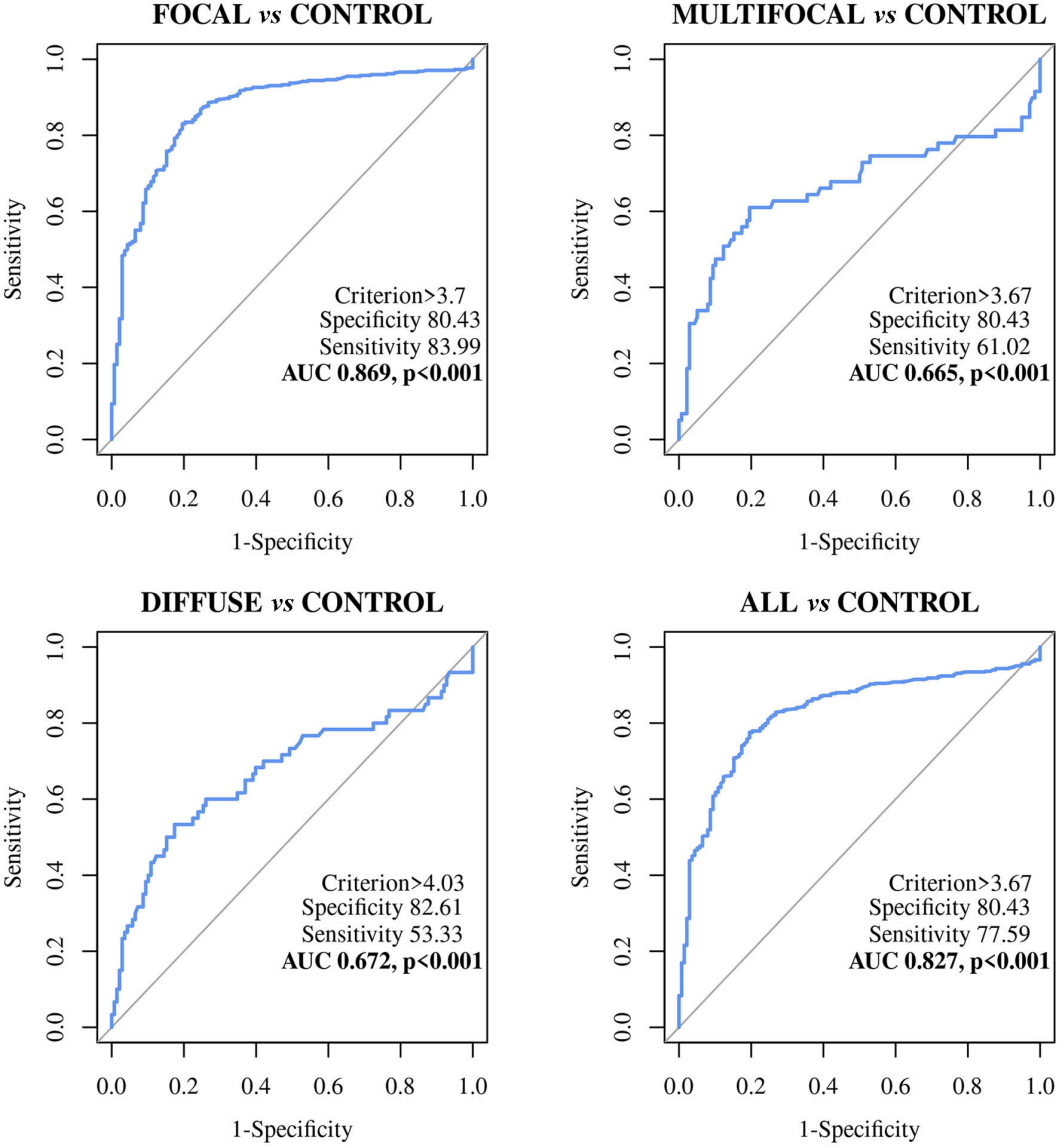
Receiver Operator Characteristic Curves (ROC curves) of the ABCA13 biomarker-based ELISA in Holstein Friesian cows with focal (*n* = 447), multifocal (*n* = 59), diffuse histological lesions (*n* = 60) and any type of paratuberculosis (PTB) lesions (*n* = 566) *vs*. control animals from PTB-free farms (*n* = 138); All, includes all animals with focal, multifocal and diffuse lesions; ABCA13, bovine ATP binding cassette subfamily A member 13.

**Figure 3:**
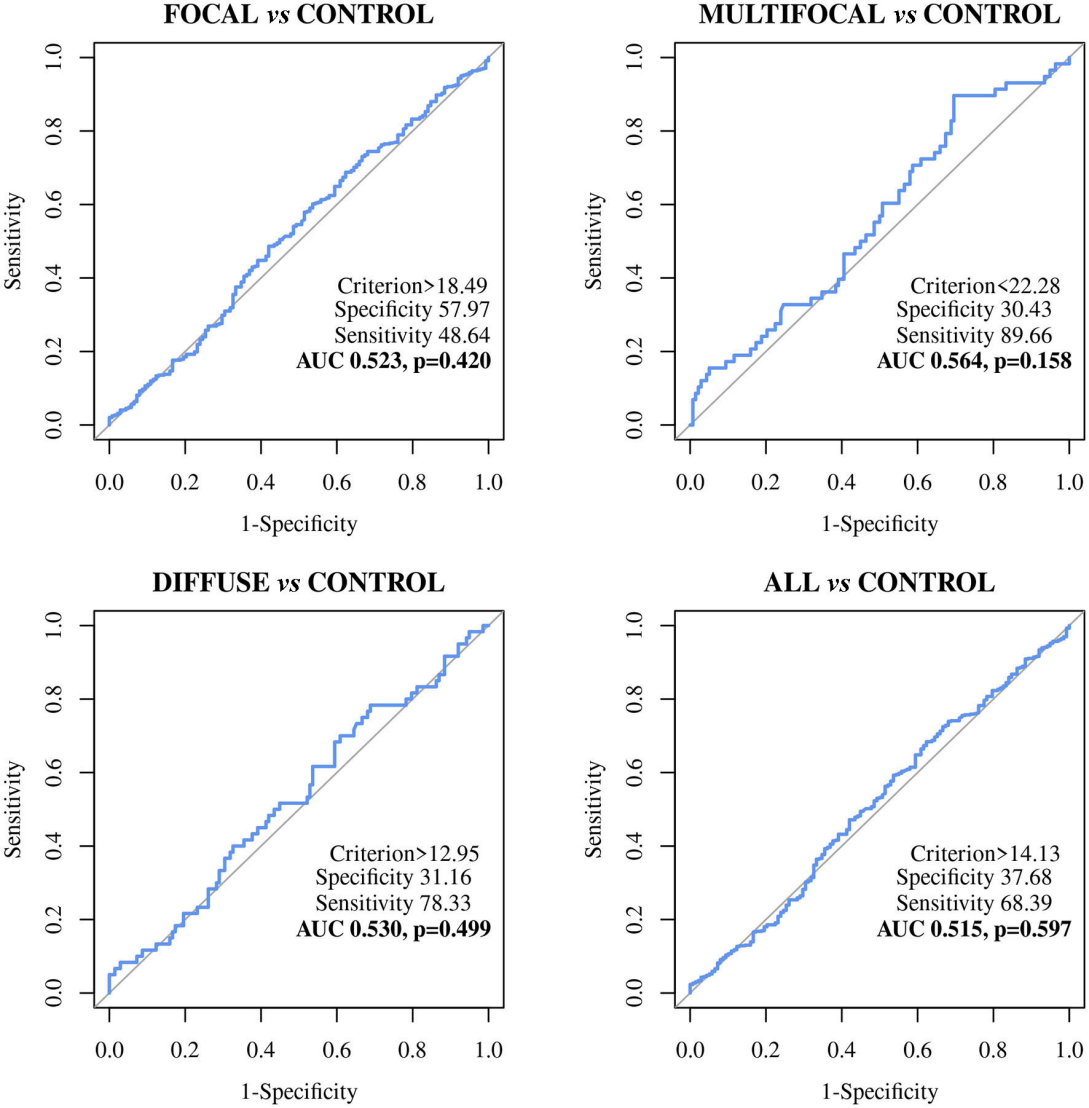
Receiver Operator Characteristic Curves (ROC curves) of the MMP8 biomarker-based ELISA in Holstein Friesian cows with focal (*n* = 442), multifocal (*n* = 58), diffuse lesions (*n* = 60) and any type of lesions (*n* = 560) *vs*. control animals from PTB-free farms (*n* = 138); All, includes all animals with focal, multifocal and diffuse lesions; MMP8, bovine matrix metallopeptidase 8.

The diagnostic performance of the ABCA13-based ELISAs was investigated by ROC analysis of the biomarker concentrations using a total of 704 sera and plasma samples, including 447 from focal animals, 59 from multifocal, 60 from diffuse, and 138 sera from control animals (PTB-free farms). ROC analysis of the MMP8-based ELISA was performed using 698 samples, 442 were from animals with focal lesions, 58 with multifocal, 60 with diffuse, and 138 from negative controls (6 sera were only analyzed by the ABCA13-based ELISA as we did not have enough volume to perform both ELISAs). The individual concentrations of ABCA13 and MMP8 in the serum of each cow are shown in Figure 1 and the mean values of the ABCA13 and MMP8 biomarkers concentrations for each group are shown in Table 2. The AUCs values, optimal cut-off points, specificities and sensitivities of each ELISA were estimated by ROC analysis for each histopathological form individually as well as for all histopathological forms globally (Table 3). ROC curves corresponding to the ABCA13 and MMP8-based ELISAs for the different histopathological groups compared to the PTB-free control group are shown in Figures 2, 3, respectively.

**Table 2:** ABCA13 and MMP8 mean concentration values in the different histopathological groups.

	**ABCA13**	**MMP8**
**paratuberculosis (PTB) histopathological groups**	**Mean ±STD**	**Mean ±STD**
Focal	8.10 ± 5.98***	23.23 ± 29.66
Multifocal	5.44 ± 4.47***	18.24 ± 13.90
Diffuse	5.05 ± 3.95***	24.96 ± 25.81
All lesion types	7.50 ± 5.77***	22.90 ± 28.05
Control	2.91 ± 2.04	20.66 ± 13.52

ABCA13, bovine ATP binding cassette subfamily A member 13; MMP8, bovine matrix metallopeptidase 8; STD, standard deviation; Control, refers to the PTB-free control group; The concentration values are expressed as ng/mL The asterisks indicate if the differences between each histopathological group and the control are or not significant (***p <0.001).

**Table 3:** Diagnostic performance of the ABCA13 and MMP8-based ELISAs for the detection of animals with different types of PTB histological lesions.

	**ELISA**	***N***	**AUC**	***p*-value**	**Cut-off**	**Se (%)**	**Sp (%)**	**DV**
FOCAL *vs*. CONTROL	ABCA13	447	0.869	<0.001	>3.7	82.99	80.43	0.822
	MMP8	442	0.523	0.420	>18.49	48.64	57.97	0.533
MULTIFOCAL *vs*. CONTROL	ABCA13	59	0.665	<0.001	>3.67	61.02	80.43	0.707
	MMP8	58	0.564	0.158	<22.28	89.66	30.43	0.600
DIFFUSE *vs*. CONTROL	ABCA13	60	0.672	<0.001	>4.03	53.33	82.61	0.680
	MMP8	60	0.530	0.499	>12.95	78.33	31.16	0.547
ALL *vs*. CONTROL	ABCA13	556	0.827	<0.001	>3.67	77.56	80.43	0.790
	MMP8	560	0.515	0.603	>14.12	68.39	37.68	0.530

*AUC, area under the curve; p-value, p-value of the AUC area, indicates whether the discrimination between animals with focal, multifocal, diffuse or with any type of lesion and the control animals is significant or not; the cut-off point is expressed as ng/mL and indicates the ABCA13 or MMP8 concentration above which an animal is considered positive; Se, sensitivity; Sp, specificity; DV, diagnostic value (semi-sum of sensitivity and specificity); vs., versus; ABCA13, bovine ATP binding cassette subfamily A member 13; MMP8, bovine matrix metallopeptidase 8; The control group consists of the 138 negative reference animals belonging to the 4 herds with a PTB-free history in the last 3 to 5 years*.

The ELISA based on the detection of the ABCA13 biomarker showed a good discriminatory power (0.8 < AUC <0.9) between animals with focal lesions and control animals with an AUC value of 0.869 [(0.836–0.903, 95% CI), *P* < 0.001], a sensitivity of the 82.99% and a specificity of 80.43%. However, its discriminatory power between animals with multifocal or diffuse lesions and the control animals was poor (AUC <0.7). Nevertheless, when it comes to differentiating between animals with any type of lesion and control animals the ABCA13-based ELISA showed a good discriminatory power with an AUC of 0.827 (0.792–0.862, 95% CI), *p* < 0.001), a sensitivity of the 77.56% and a specificity of the 80.43%. Global detection improves as a consequence of the good performance of the ELISA for detection of animals with focal lesions, which are more numerous in the analysis of the group including all the lesion types.

The MMP8-based ELISA showed a poor discriminatory power between the different histopathological groups and the control animals. The AUC values were <0.7 in all cases (Table 3) with the sensitivities ranging from 48.64% to 89.66%, the specificities from 26.09% to 57.97%, and the diagnostic values from 0.530 to 0.6.

### Comparison of the Diagnostic Performance of the ABCA13-Based ELISA With That of Other Conventional Methods

Given the good discriminatory power of the ABCA13-based ELISA its diagnostic performance was compared with that of other conventional PTB diagnostic methods (IDEXX ELISA for serum detection of Map-specific antibodies, PCR and bacteriological culture of feces and tissues) and the Ziehl-Neelsen staining (see Table 4). The MMP8-based ELISA was not compared with the conventional methods due to its low diagnostic performance. The ABCA13-based ELISA showed a higher diagnostic value (semi-sum of sensitivity and specificity) (0.822) than the IDEXX ELISA (0.517), the fecal bacteriological culture (0.523), and real-time PCR (0.531) for the detection of animals with focal lesions. In the same way, this biomarker-based ELISA had the best diagnostic value for detection of animals with multifocal lesions (0.707) and for the global detection of animals with any type of lesions (0.790) when compared with the above-mentioned techniques. However, when it comes to the detection of animals with diffuse lesions the method that presented the best diagnostic value was the conventional IDEXX ELISA (0.933). The combined use of the ABCA13-based ELISA and the IDEXX ELISA would increase sensitivity from 77.66% to 83.03% as the IDEXX ELISA detected 31 animals (3 focal, 4 multifocal, and 24 diffuse) that were not detected by the ABCA13 ELISA. Regarding specificity, the IDEXX ELISA performance was better. The ABCA13 ELISA detected 27 control animals as positive (80.43% specificity) while the IDEXX ELISA detected the 138 negative control animals as negative (100% specificity).

**Table 4:** Comparison of the diagnostic performance of the ABCA13-based ELISA with that of other conventional paratuberculosis (PTB) diagnostic methods.

**Method**	**Se (%)**	**Sp (%)**	**DV**	**Time to eradication**	**Benefit/cost 20 years**
**Detection animals with focal lesions (*****n*** **=** **447)**					
**ABCA13 ELISA**	**82.99**	**80.43**	**0.822**		
IDEXX ELISA	3.36	100	0.517		
Z-N	17.08	/	/		
Fecal real-time PCR	6.15	100	0.531		
Tissues real-time PCR	16.85	/	/		
Fecal culture	4.65	100	0.523		
Tissues culture	15.51	/	/		
**Detection animals with multifocal lesions (*****n*** **=** **59)**					
**ABCA13 ELISA**	**61.02**	**80.43**	**0.707**		
IDEXX ELISA	30.51	100	0.653		
Z-N	77.59	/	/		
Fecal real-time PCR	40	100	0.7		
Tissues real-time PCR	59.32	/	/		
Fecal culture	12	100	0.560		
Tissues culture	50.85	/	/		
**Detection animals with diffuse lesions (*****n*** **=** **60)**					
ABCA13 ELISA	53.33	82.61	0.680		
**IDEXX ELISA**	**86.67**	**100**	**0.933**		
Z-N	100	/	/		
Fecal real-time PCR	77.27	100	0.886		
Tissues real-time PCR	90	/	/		
Fecal culture	13.64	100	0.568		
Tissues culture	85	/	/		
**Detection animals with any type of lesion (*****n*** **=** **566)**					
**ABCA13 ELISA (Cut-off: 3.7)**	**77.03**	**80.43**	**0.790**	**6 years**	**0.46**
ABCA13 ELISA (Cut-off: 7.0)	42.40	97.10	0.787	14 years	0.90
IDEXX ELISA	15.02	100	0.575	>20 years	0.98
Z-N	32.03	/	/	/	/
Fecal real-time PCR	31.63	100	0.658	>20 years	1.07
Tissues real-time PCR	29.08	/	/	/	/
Fecal culture	9.18	100	0.546	>20 years	0.80
Tissues culture	26.6	/	/	/	/

*Se, sensitivity; Sp, specificity; DV, diagnostic value (semi-sum of sensitivity and specificity); Z-N, Ziehl-Neelsen staining; PCR (polymerase chain reaction); /, no data available. The estimation of the specificity for these methods is based on 138 negative control animals for the IDEXX ELISA while for the fecal real time PCR and fecal bacteriological culture is based in the animals of one of the PTB-free farms (n = 61). The specificity could not be estimated for the tissue-dependent techniques as in the control animals we are working with live animals. The diagnostic methods with the best diagnostic value for each of the groups are shown in bold. Simulation conditions: Herd of 200 cows with 40% infection rate and 5% clinical cases; Testing costs 15€/sample; One testing/year; Loss by death 1,000€, by culling 500€; Increased value of cows for herd freedom from PTB 50% higher*.

In addition, the performance of each diagnostic test was compared in a 20 year dynamics simulation study of low level PTB clinical incidence on a cattle farm of 200 heads (Table 4) with the Juste & Casal model (83). It can be observed that the use of the optimal cut-off value of the ABCA13 ELISA (3.67 ng/mL) with the higher diagnostic value would allow an advance in infection eradication to 6 years from program start (>70% reduction) with just an 54% decrease in the benefit/cost ratio (0.45 *vs*. 0.98) compared to the antibody ELISA. Using a more conservative cut-off value of 7 ng/mL (obtained as the mean value of negative control group + 2 standard deviations (84), characterized by a sensitivity of 42.40% and a higher specificity of 97.1%), it would be possible to eradicate the infection in 14 years (>30% reduction) with an 8% lower benefit/cost ratio (0.90).

### Assessment of Cross-Reactivity of the ABCA13-Based ELISA With Sera From Animals Infected With *Mycobacterium bovis*

The cross-reactivity of the ABCA-13-based ELISA was assessed using sera and plasma samples from cows experimentally (*n* = 30) infected with *M. bovis* and confirmed natural cases detected in the national bovine TB eradication program (*n* = 27). No cross-reactivity with sera and plasma samples of experimentally infected animals was detected (30 out of 30 samples were negative), however, the ABCA13-based ELISA showed some degree of crossed-reactivity with sera from natural cases (7 samples out of 27 cross-reacted with ABCA13, 25.92%).

## Discussion

The goals of PTB control programs vary from eradication in areas of low prevalence, to control in areas with high prevalence, and increased surveillance in areas with no prior history of disease (85). Early detection of Map subclinical infections is critical for PTB control; however, current diagnostic tools have low sensitivities for the detection of subclinically infected animals. Host biomarkers may provide improved diagnostic tools for PTB increasing the effectiveness of control programs. In the present study, we performed a robust validation of ELISAs based on the detection of two candidate biomarkers using a large collection of serum samples (*n* = 704) from positive and negative reference animals. Evaluation of these two biomarkers showed the potential of one of them, the ABCA13, for detection of most histopathological forms of bovine PTB while the other, the MMP8, was ruled out due to its low sensitivity. Animals, studied at the end of their productive life, were initially classified according to the different pathological forms associated with Map infection (uninfected, focal, multifocal and diffuse lesions), and thus avoided the high rate of false negative results yielded by the most used reference immunological and microbiological methods. The unexpected shortcoming of the pathological characterization was the low number of uninfected negative animals found among these animals. This led to a search for a set of reference negative sera characterized by other, more readily implemented, methods. This was done by reviewing records of PTB serological testing from Asturian farms voluntarily engaged in an animal health program. Finally, a reasonable number of farms with between 3 and 5 years of whole herd negative follow-up was gathered as the largest number of negative samples available. These criteria were not as rigorous as histopathological examination, but by way of repeated whole herd negative results, and absence of clinical cases, provided the closest approximation that allows an estimation of specificity in natural conditions.

The MMP8-based ELISA had a poor discriminatory power (AUC values <0.7) between the different histopathological groups and the control animals (AUCs ranging from 0.515 to 0.564) making it a poor candidate to its use as a PTB diagnostic biomarker. The percentage of positivity detected by the MMP8-based ELISA and the serum levels of this protein varied only slightly between the different histopathological groups (Figure 1 and Table 2), and no significant differences in the serum levels were detected between the different histological groups and the control. These results were in disagreement with those found in the RNA-Seq analysis (35) and the pre-validation study (36) underlying the fact that correlations between RNA expression and protein levels are not always strong (86) and the importance of the current study, based on the analysis of a large number of samples, to verify the potential of putative novel biomarkers.

The ABCA13 ELISA had a good discriminatory power (0.8 ≤ AUC <0.9, *p* < 0.001) between animals with focal lesions, animals with any type of lesion, and the control animals from PTB-free farms. Furthermore, the ABCA13-based ELISA had higher sensitivities and diagnostic values than the IDEXX ELISA and the fecal and tissue bacteriological isolation and real-time PCR for the detection of animals with focal, multifocal lesions and those with any type of lesion. More specifically, the ELISA based on the detection of ABCA13 had the best diagnostic performance for the detection of animals with focal lesions and for global detection of animals with any type of lesions, being capable of detecting 83.99 and 77.59% of these animals, respectively. However, the IDEXX ELISA, fecal PCR and fecal culture only detected 3.36%, 6.15%, and 4.65% animals with focal lesions and 15.02, 31.63, and 9.18% of the animals with any type of lesion, respectively. The ABCA13-based ELISA specificity values ranged between 80.43 and 82.61%, however, the IDEXX ELISA, the fecal PCR and fecal bacteriological culture had better specificity (100%). These relatively low values of specificity as well as the high rate of positives in the natural TB cases indicate that the high sensitivity of this ELISA might be counterbalanced by some degree of unspecific reactivity due to causes other than PTB. However, it cannot be ruled out that this lower specificity performance could be due to the existence of infected animals in the PTB-free farms that have not been recognized due to the low sensitivity of the reference criteria (IDEXX ELISA) (87) used for the determination of the PTB-infectious status of the negative control animals. It should be pointed out that these sensitivity and specificity values correspond to the optimal cut-off value estimated by ROC analysis. However, the quantitative nature of the protocol reported here becomes a strength since it could be adapted to each specific epidemiological and economic circumstance. For instance, simply by raising the cut-off to 7.0 ng/mL (the upper 99.5% ABCA13 concentration of the negative control group), specificity can become 97.10% still keeping a 42.40% sensitivity that is more than twice that of the antibody IDEXX ELISA (Table 4). The simulation study showed that the ABCA13 ELISA increase in sensitivity could greatly improve the chances of reaching PTB eradication in a reasonable period of time. Even though the benefit/cost would still be negative and therefore, less attractive than vaccination (83), this method would provide a new alternative to farmers or animal health managers to quickly bring under control any outbreak of PTB.

The study of the cross-reactivity of the ABCA13-based ELISA with sera from experimentally and naturally infected *M. bovis* cows showed that young experimentally infected animals with a negative background do not cross-react. However, 25.92% of the naturally infected animals did. This cross-reactivity migh be due to the uncontrolled history of these animals where exposure to other mycobacteria cannot be ruled out. Moreover, it is also possible that the animals included in this group, which are negative by IDEXX ELISA, are not detected as positive for PTB because of the low sensitivity of the IDEXX ELISA to detect animals with focal lesions. Further studies will be performed to asses potential cross-reactions with other micobacteria or pathogens.

The percentage of positive animals in the ABCA13-based ELISA varied between the different pathological forms. The percentage of animals with a positive ABCA13 result was higher in animals with focal lesions, followed by animals with multifocal and diffuse lesions (83.99%, 61.02% and 53.33%, respectively). Likewise, the individual mean expression levels of ABCA13 in the focal, multifocal and diffuse groups was significantly higher than in the control group (see Figure 1, Table 2). This expression pattern across pathological forms was similar to that observed for intelectin 2 (ITLN2) in the ileocecal valve (ICV) in a recently published study (81). These findings validated our initial study where RNA-seq results showed that the ABCA13 and ITLN2 genes were overexpressed in peripheral blood (log2 fold change = 3.7) and ICV (log2 fold change 10.6) of animals with focal lesions compared to control animals without lesions (35). In fact, the precursor genes of ABCA13 and ITLN2, were the most overexpressed genes in peripheral blood and ICV of animals with focal lesions, respectively.

As we have previously mentioned, the aim of this study was to carry out a broad-scale validation of the ABCA13-based ELISA (*n* = 704) to confirm the potential of ABCA13 as a diagnostic biomarker. Animals with focal lesions very often go unnoticed in infected farms due to the low sensitivity of conventional methods to detect this group of animals (Table 4). To this goal, ABCA13-based ELISA could be a useful diagnostic tool for the detection of animals with focal lesions, thus complementing conventional methods that have a better diagnostic performance on the diffuse lesions group (Table 4).

The ELISA based on the ABCA13 biomarker has proven to be useful for global diagnosis of animals with lesions associated with PTB, although its diagnostic performance is not good in the case of animals with diffuse lesions. Thus, it could be considered a new tool in the diagnosis of Map infections, either individually, or in combination with other conventional diagnostic methods that detect animals with diffuse lesions accurately as is the case of the IDEXX ELISA. With respect to this, the IDEXX ELISA is very sensitive to detect animals with diffuse lesions, further supporting the idea of using the ABCA13-based ELISA and the IDEXX ELISA in combination, which would increase sensitivity from 77.66% to 83.03% as the IDEXX ELISA detected 31 animals (3 focal, 4 multifocal, and 24 diffuse) that were not detected by the ABCA13 ELISA. Future studies will be focus on validating the assay in younger cohorts as detection of positive animals or selection of negative ones will be more useful at early stages in their life.

Similar studies using biomarkers, but evaluated in a smaller number of animals, have been described by other authors. Espinosa et al. (88) assessed the serum levels of haptoglobin and serum amyloid A, two inflammatory acute-phase proteins detected during the course of PTB infection, in 190 PTB naturally infected animals classified according to the different pathological forms associated with infection (59 uninfected animals without lesions, 73 with focal lesions, 19 with multifocal, 11 with diffuse paucibacillary, and 28 with diffuse multibacillary lesions). Their results reflected a significant increase in the levels of these proteins in the infected animals compared to the control group, more specifically, in those with types of lesions characterized by a low bacterial load and with predominance of a cell-mediated immune response. The authors concluded that these molecules could show potential as putative biomarkers of PTB infection, especially for identification of subclinical animals showing pathological forms related to latency or resistance to the development of advance lesions. Park et al. (89) identified alpha-2-macroglobulin (A2M) as a new promising biomarker improving the diagnostic sensitivity of bovine PTB. They showed that serum A2M levels were significantly higher in Map-exposed (*n* = 20), subclinical shedder (*n* = 27), subclinical non-shedder (*n* = 50) and clinical shedder (*n* = 18) groups that in the healthy control group (*n* = 11, from a PTB free farm, negative by fecal PCR and IDEXX and ID Vet ELISA). A2M ELISA showed superior diagnostic performance (90.4 sensitivity and 100% specificity) than other biomarkers and two commercial ELISAs for detection of anti-Map antibodies although the number of healthy control animals included in the study to estimate the specificity is quite small due to difficulty finding a PTB-free farm.

In summary, we could conclude that the ELISA based on the detection of the ABCA13 biomarker has potential for use as a new diagnostic tool for the detection of subclinical animals, which would allow the establishment of an adequate management protocol on farms that, together with hygienic-sanitary measures, could help to reduce the incidence of PTB and, ultimately, reduce the prevalence of this costly and widespread disease. As PTB has a long incubation period before disease becomes evident, early diagnosis of subclinical animals is important to control the spread of the disease.Conversely, we could rule out the use of the ELISA, based on the detection of the MMP8 protein as a diagnostic tool for the detection of Map infection.

## Data Availability Statement

The datasets presented in this study can be found in online repositories. The names of the repository/repositories and accession number(s) can be found at: NCBI Gene Expression Omnibus (GEO) database under the accession number GSE137395.

## Ethics Statement

The animal study was reviewed and approved the SERIDA Animal Ethics Committee and authorized by the Regional Consejería de Agroganadería y Recursos Autóctonos del Principado de Asturias –Spain- (authorization codes PROAE 29/2015 and PROAE 66/2019). All the procedures were carried out in accordance with Directive 2012/63/EU of the European Parliament and the Spanish RD53/2013. Peripheral blood and fecal samples were collected by trained personnel and in accordance with good veterinary practice. Written informed consent was obtained from the owners for the participation of their animals in this study.

## Author Contributions

RC and MA-H: conceptualization and funding acquisition. CB-V and NI: data curation. RAJ, TI, and RC: formal analysis. CB-V, RAJ, JMG, AB, MC, and RC: investigation. CB-V, NI, JA, PV, MC, and TI: methodology. PV, AB, and MQ: resources. RAJ and TI: validation. CB-V and RC: writing—original draft. CB-V, MA-H, NI, PV, RAJ, JMG, AB, MC, JA, MAQ, TI, and RC: writing—review and editing. All authors contributed to the article and approved the submitted version.

## Funding

This study was part of the I+D+i project (RTI2018-094192-R-C22) and was funded by the Spanish MCIN/AEI/10.13039/501100011033/ Ministry of Science, Innovation and the European Regional Development Funds (FEDER Una manera de hacer Europa) and by the Gobierno del Principado de Asturias, Regional funds PCTI 2021–2023 (GRUPIN: IDI2021-000102) co-funded by FEDER. We acknowledge the National Institute for Agricultural Research (INIA) for the scholarships of CB-V (Ayuda CPD2016-0142 financiada por MCIN/AEI/ 10.13039/501100011033 y FSE El FSE invierte en tu future) and MC.

## Conflict of Interest

The authors declare that the research was conducted in the absence of any commercial or financial relationships that could be construed as a potential conflict of interest.

## Publisher's Note

All claims expressed in this article are solely those of the authors and do not necessarily represent those of their affiliated organizations, or those of the publisher, the editors and the reviewers. Any product that may be evaluated in this article, or claim that may be made by its manufacturer, is not guaranteed or endorsed by the publisher.

## Supplementary Material

Supplementary Figure 1 Histological characterization of bovine tissue samples. Animals were classified according to the type of PTB- associated histological lesions present in their gut tissues and associated lymph nodes after staining with hematoxylin-eosin **(A–F)** and Ziehl-Neelsen **(G)**. **(A)** Animal with no lesions; **(B)** Focal lesion in ileocecal lymph node. Small and focal granulomas (arrows) consisting of macrophages and lymphocytes are observed in the Peyer's patch; **(C)** Multifocal lesion in the ileocecal valve. Small granulomas (arrows) consisting of macrophages and lymphocytes are present in the intestinal mucosa as well as Langhans multinucleated giant cells (star) isolated or associated with these granulomas; **(D)** Paucibacillary diffuse lesion in distal jejunum. Diffuse granulomatous enteritis with lymphocyte-predominant inflammatory infiltrate. A granuloma in the intestinal mucosa (arrow) consisting of a small number of macrophages and multinucleated giant cells can be observed. An isolated giant cell is also observed (star); **(E)** Intermediated diffuse lesion in distal jejunum. The infiltrate consists of lymphocytes, plasma cells and a moderate number of macrophages. Granulomas are observed (arrows); **(F)** Multibacillary diffuse lesion in ileocecal valve. The infiltrated consists of macrophages within a large number of acid-alcohol resistant bacilli to Ziehl-Neelsen staining **(G)**. **(A–F)** Bar = 100 microns and **(G)** Bar = 50 microns.
