# Supplementary figures and images for: Use of ATP-Binding Cassette Subfamily A Member 13 (ABCA13) for Sensitive Detection of Focal Pathological Forms of Subclinical Bovine Paratuberculosis
Source: Front Vet Sci. 2022 Mar 10;9:816135. doi: 10.3389/fvets.2022.816135 (PMC8960928; doi:10.3389/fvets.2022.816135)

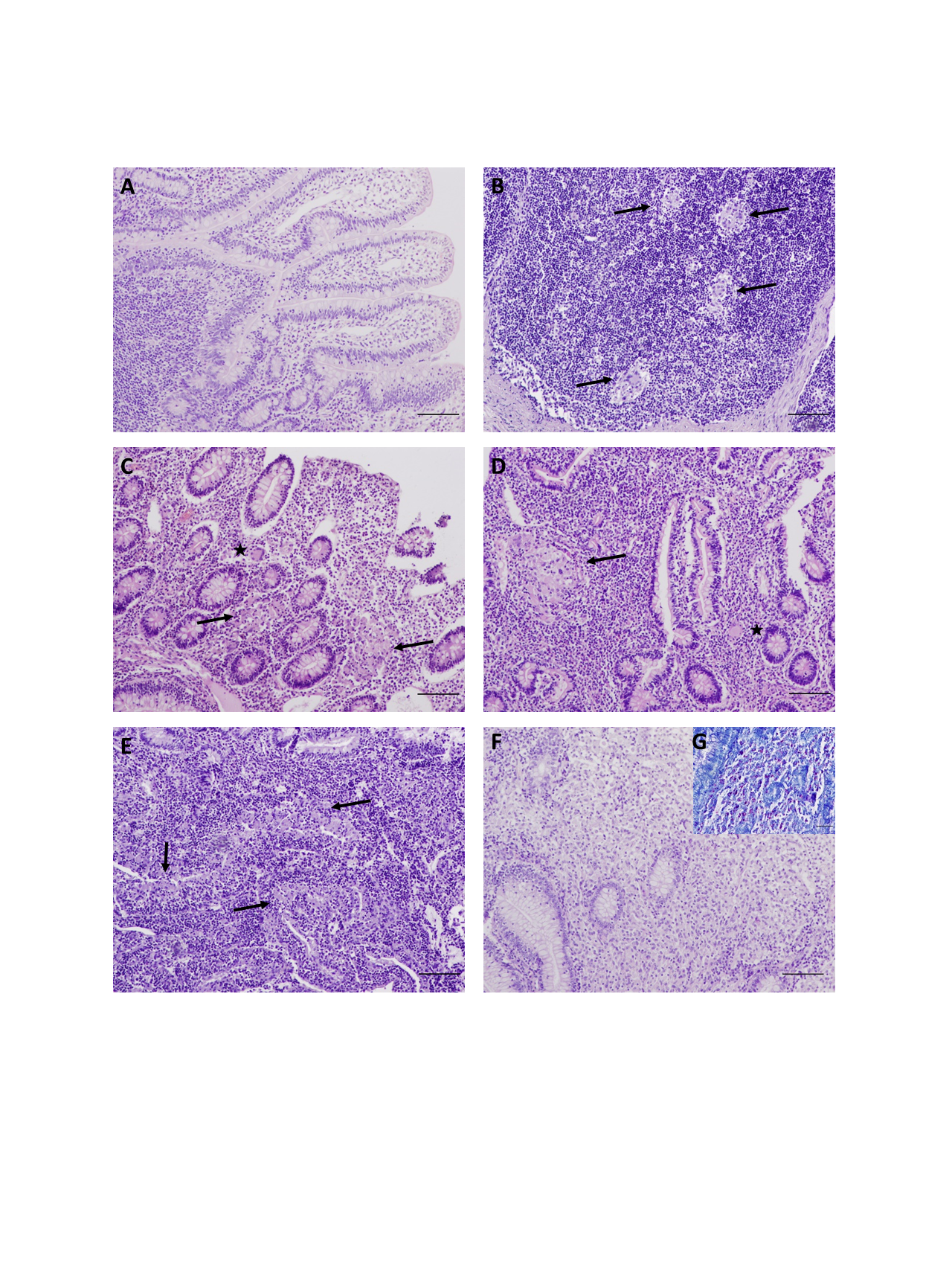

Supplement: Supplementary Figure 1 — Histological characterization of bovine tissue samples. Animals were classified according to the type of PTB- associated histological lesions present in their gut tissues and associated lymph nodes after staining with hematoxylin-eosin (A–F) and Ziehl-Neelsen (G). (A) Animal with no lesions; (B) Focal lesion in ileocecal lymph node. Small and focal granulomas (arrows) consisting of macrophages and lymphocytes are observed in the Peyer's patch; (C) Multifocal lesion in the ileocecal valve. Small granulomas (arrows) consisting of macrophages and lymphocytes are present in the intestinal mucosa as well as Langhans multinucleated giant cells (star) isolated or associated with these granulomas; (D) Paucibacillary diffuse lesion in distal jejunum. Diffuse granulomatous enteritis with lymphocyte-predominant inflammatory infiltrate. A granuloma in the intestinal mucosa (arrow) consisting of a small number of macrophages and multinucleated giant cells can be observed. An isolated giant cell is also observed (star); (E) Intermediated diffuse lesion in distal jejunum. The infiltrate consists of lymphocytes, plasma cells and a moderate number of macrophages. Granulomas are observed (arrows); (F) Multibacillary diffuse lesion in ileocecal valve. The infiltrated consists of macrophages within a large number of acid-alcohol resistant bacilli to Ziehl-Neelsen staining (G). (A–F) Bar = 100 microns and (G) Bar = 50 microns. [file Image_1.TIF]
